# Author Correction: Nutrients cause consolidation of soil carbon flux to small proportion of bacterial community

**DOI:** 10.1038/s41467-021-24314-2

**Published:** 2021-06-24

**Authors:** Bram W. Stone, Junhui Li, Benjamin J. Koch, Steven J. Blazewicz, Paul Dijkstra, Michaela Hayer, Kirsten S. Hofmockel, Xiao-Jun Allen Liu, Rebecca L. Mau, Ember M. Morrissey, Jennifer Pett-Ridge, Egbert Schwartz, Bruce A. Hungate

**Affiliations:** 1 Earth and Biological Sciences Directorate, Pacific Northwest National Laboratory, Richland, WA USA; 2 Center for Ecosystem Science and Society, Northern Arizona University, Flagstaff, AZ USA; 3 Department of Biological Sciences, Northern Arizona University, Flagstaff, AZ USA; 4 Physical and Life Sciences Directorate, Lawrence Livermore National Laboratory, Livermore, CA USA; 5 Department of Agronomy, Iowa State University, Ames, IA USA; 6 Institute for Environmental Genomics, Department of Microbiology and Plant Biology, University of Oklahoma, Norman, OK USA; 7 Pathogen and Microbiome Institute, Northern Arizona University, Flagstaff, AZ USA; 8 Division of Plant and Soil Sciences, West Virginia University, Morgantown, WV USA; 9 Life and Environmental Sciences Department, University of California Merced, Merced, CA USA

**Keywords:** Carbon cycle, Microbial ecology, Carbon cycle

Correction to: *Nature Communications* 10.1038/s41467-021-23676-x, published online 7 June 2021.

The original version of this Article contained an error in the author affiliations.

The affiliation of Jennifer Pett-Ridge with Life and Environmental Sciences Department, University of California Merced, Merced, CA was inadvertently omitted.

This has now been corrected in both the PDF and HTML versions of the Article.

