# Supplementary material for: Nutrients cause consolidation of soil carbon flux to small proportion of bacterial community
Source: Nat Commun. 2021 Jun 7;12:3381. doi: 10.1038/s41467-021-23676-x (PMC8184982; doi:10.1038/s41467-021-23676-x)
Supplement: Supplementary file 1 — Supplementary Information [file 41467_2021_23676_MOESM1_ESM.pdf]

Supplemental Information for:

Nutrients cause consolidation of soil carbon flux to small proportion of bacterial community

Bram W Stone, Junhui Li, Benjamin J Koch, Steven J Blazewicz, Paul Dijkstra, Michaela Hayer, Kirsten S Hofmockel, Xiao-Jun Allen Liu, Rebecca L Mau, Ember Morrissey, Jennifer Pett-Ridge, Egbert Schwartz, Bruce A Hungate

Nature Communications

Corresponding author: Bram W Stone bram.stone@pnnl.gov

Supplementary Tables

	Mixed Conifer forest			Ponderosa Pine forest			Piñon pine – Juniper scrubland			Desert grassland		
	**Genus**	**Flux**	**Abund**	**Genus**	**Flux**	**Abund**	**Genus**	**Flux**	**Abund**	**Genus**	**Flux**	**Abund**
**Control**	*Bradyrhizobium*	25.0	8.79	*Bradyrhizobium*	21.8	5.61	*RB41*	29.6	12.1	*Bradyrhizobium*	42.3	14.1
	RB41	12.6	5.39	*RB41*	17.6	6.36	*Bradyrhizobium*	10.9	3.39	*RB41*	10.8	5.54
	Haliangium	7.63	2.36	*Haliangium*	3.59	2.88	*Amycolatopsis*	4.32	0.71	*Streptomyces*	2.80	4.12
	Solirubrobacter	3.23	3.30	*Gemmatimonas*	2.67	4.97	*Streptomyces*	2.26	2.94	*Solibrubrobacter*	2.67	4.29
	Gaiella	2.51	1.84	*Streptomyces*	2.50	2.47	*Bryobacter*	1.81	1.49	*Blastocatella*	1.83	1.13
**C**	*Bradyrhizobium*	23.8	5.74	*Bradyrhizobium*	26.5	7.85	*Bradyrhizobium*	23.9	8.75	*Bradyrhizobium*	18.5	3.62
	RB41	12.3	7.22	*RB41*	16.5	4.93	*RB41*	20.9	6.44	*RB41*	14.4	3.24
	Solirubrobacter	5.14	4.22	*Kribbella*	5.32	1.02	*Streptomyces*	7.00	11.3	*Streptomyces*	7.71	4.48
	Gaiella	3.20	3.32	*Solirubrobacter*	2.61	1.98	*Solirubrobacter*	3.24	3.57	*Solirubrobacter*	4.13	2.52
	Dactlyosporangium	1.99	0.33	*Pseudonocardia*	2.27	1.61	*Amycolatopsis*	2.50	0.44	*Microvirga*	2.66	1.16
**C + N**	*Bradyrhizobium*	35.5	11.9	*Bradyrhizobium*	18.9	6.99	*Streptoymces*	28.1	15.7	*Streptoymces*	53.0	18.8
	RB41	12.7	5.69	*Kribbella*	14.2	2.25	*RB41*	21.0	4.72	*Bradyrhizobium*	23.0	8.49
	Solirubrobacter	3.45	2.31	*Brk-Cab-Parabrk*	11.0	2.63	*Bradyrhizobium*	16.4	5.87	*Kribbella*	3.22	1.09
	Streptomyces	2.83	2.27	*RB41*	9.10	4.51	*Kribbella*	16.1	2.14	*RB41*	2.81	2.57
	Lysobacter	2.41	1.26	*Streptomyces*	5.65	3.77	*Solirubrobacter*	1.90	1.29	*Lechevalieria*	1.83	0.87

Supplemental Table 1 Contributions of individual bacterial genera to soil carbon flux. Contributions for each genera represent averages across three soil replicates per ecosystem (top) and soil treatment (left; Control = no amendment, C = glucose, C + N = glucose and $[NH_4]_2SO_4$). Genera are ranked from highest carbon flux to lowest for each ecosystem and treatment. For each bacterial genera, soil carbon flux calculated as the sum of productivity and respiration, estimated by ^{18}O enrichment and 16S gene abundance per gram of dry soil. Flux represents percent soil carbon flux. Abund represents relative 16S abundance (expressed as a percent). Genera that are highlighted represent common lineages across all soils and treatments. The *Brk-Cab-Parabrk* genus is an abbreviation for the *Burkholderia-Caballeronia-Paraburkholderia* group.

Primer set	Name	Sequence (5' - 3')
qPCR	*16S*-515F	AATGATACGGCGACCACCGAGTGCCAGCMGCCGCGGTAA
qPCR	*16S*-806R	CAAGCAGAAGACGGCATACGAGGACTACVSGGGTATCTAAT
Sequencing	*16S*-515F	GTGYCAGCMGCCGCGGTAA
Sequencing	*16S*-806R	GGACTACNVGGGTWTCTAAT

Supplementary Table 2 Description of primer sequences used in the amplification and sequencing of the bacterial 16S V4 region for quantitative stable isotope probing. The "Primer set" column identifies the purpose of primer groups (1 forward, 1 reverse) either for quantification of bacterial abundance through qPCR or through description of bacterial communities through Illumina MiSeq Sequencing. The "Name" column indicates the amplification target (gene, position, and direction).

Supplemental Figures

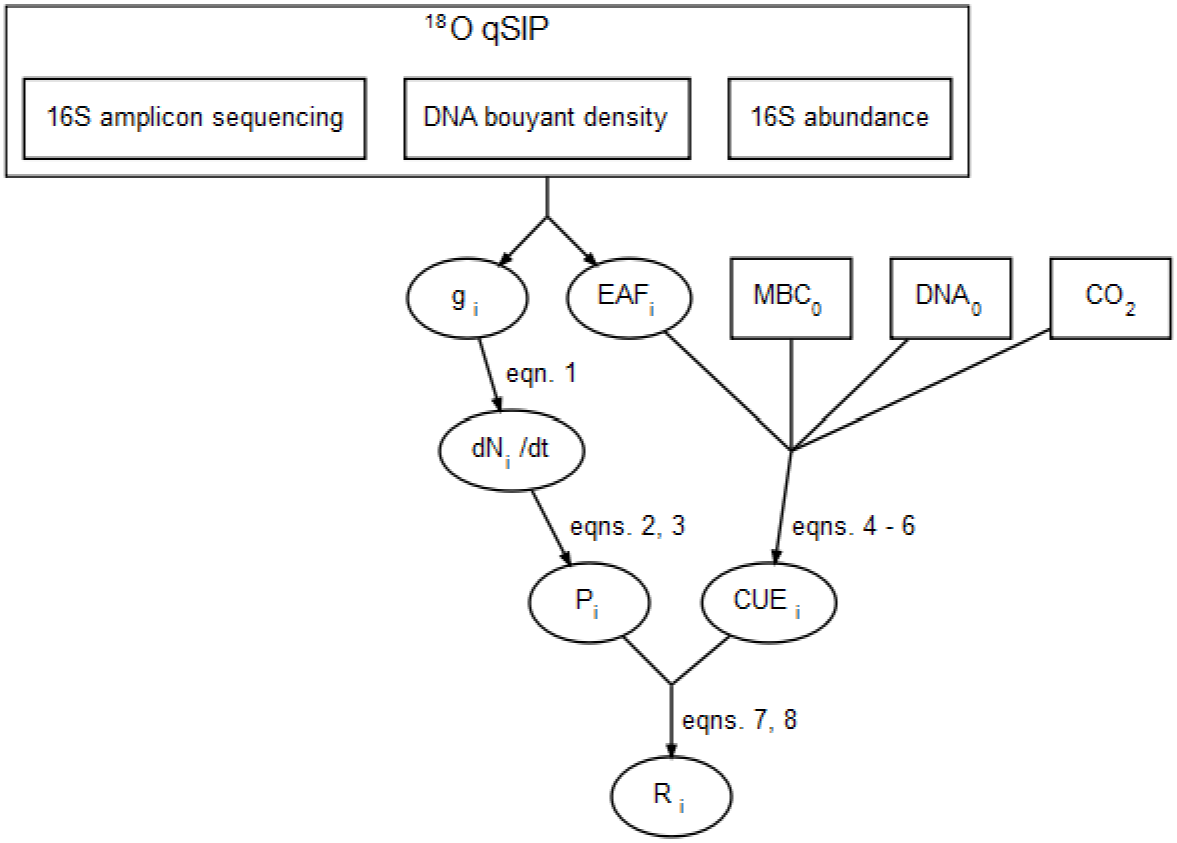

Supplemental Figure 1 Conceptual diagram for calculation of per-taxon respiration of soil bacteria. Boxes represent measured variables while ovals represent estimated values. Subscripts of 0 represent values measured, per-sample, prior to incubation (i.e., at timepoint 0). Subscripts of i represent values estimated for each bacterial taxon in a sample (enumerated by *i*). *g* and *EAF* indicate estimated per-capita growth rates and excess atom fraction, respectively, as informed by quantitative stable isotope probing (qSIP) using ^{18}O-labeled water. *MBC* and *DNA* indicate microbial biomass carbon and DNA concentration per gram of dry soil (g ds-1). CO_2 indicates C-CO_2 production (µg g ds^{-1} $week^{-1}$). *dN / dt* indicates production of *16S* copies per gram of dry soil per week. *P* indicates MBC production (µg g ds^{-1} $week^{-1}$). *CUE* indicates carbon use efficiency. *R* indicates C-CO_2 produced (µg g ds^{-1} $week^{-1}$). Labeled arrows represent corresponding equations described in the methods section. Unlabeled arrows connecting qSIP output to *g* and *EAF* represent equations not described in this text. Converging arrows represent multiple values used together in equations 4 - 6.

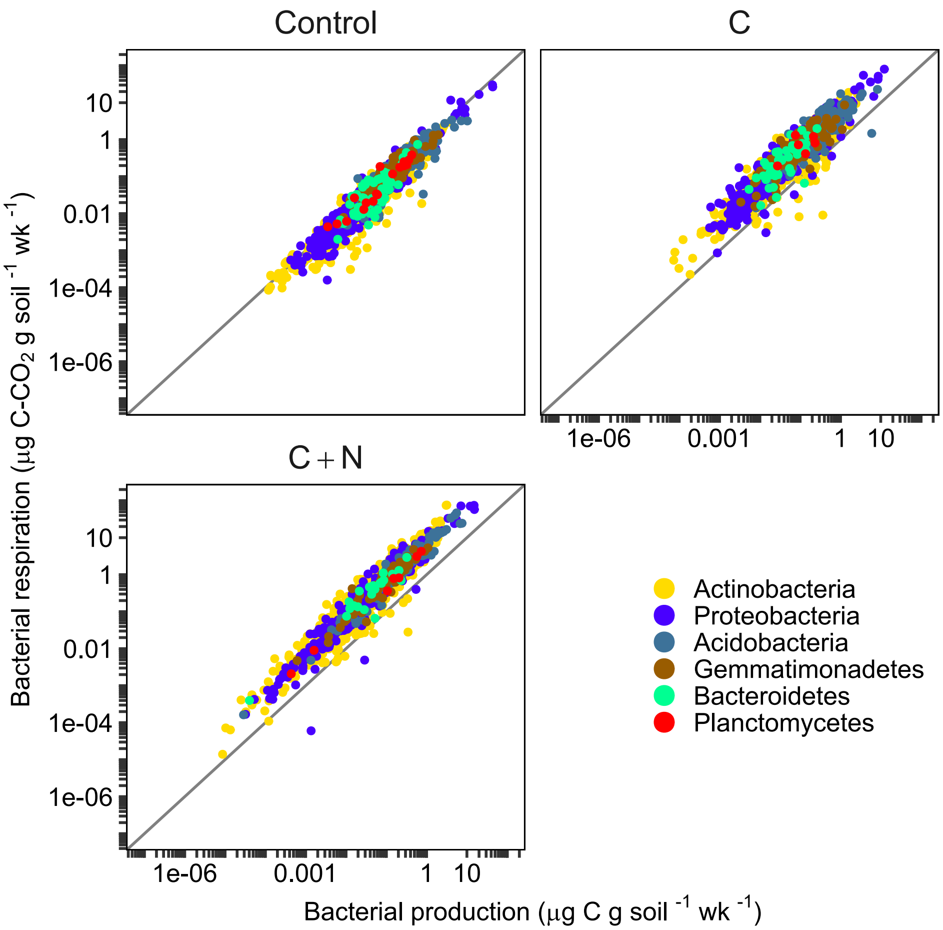

Supplementary Figure 2 Comparison of modeled bacterial productivity and respiration. Points show contributions from individual amplicon sequence variants towards modeled respiration and productivity. Results are restricted to the six bacterial phyla that cumulatively contribute to > 99% of modeled bacterial C flux. Values are estimated from soil replicates from four ecosystems (mixed conifer forest, ponderosa pine forest, piñon pine-juniper scrubland, and desert grassland) and amended with either water (labeled with Control), carbon (glucose), or carbon and nitrogen ($[NH_4]_2SO_4$) (n = 3).

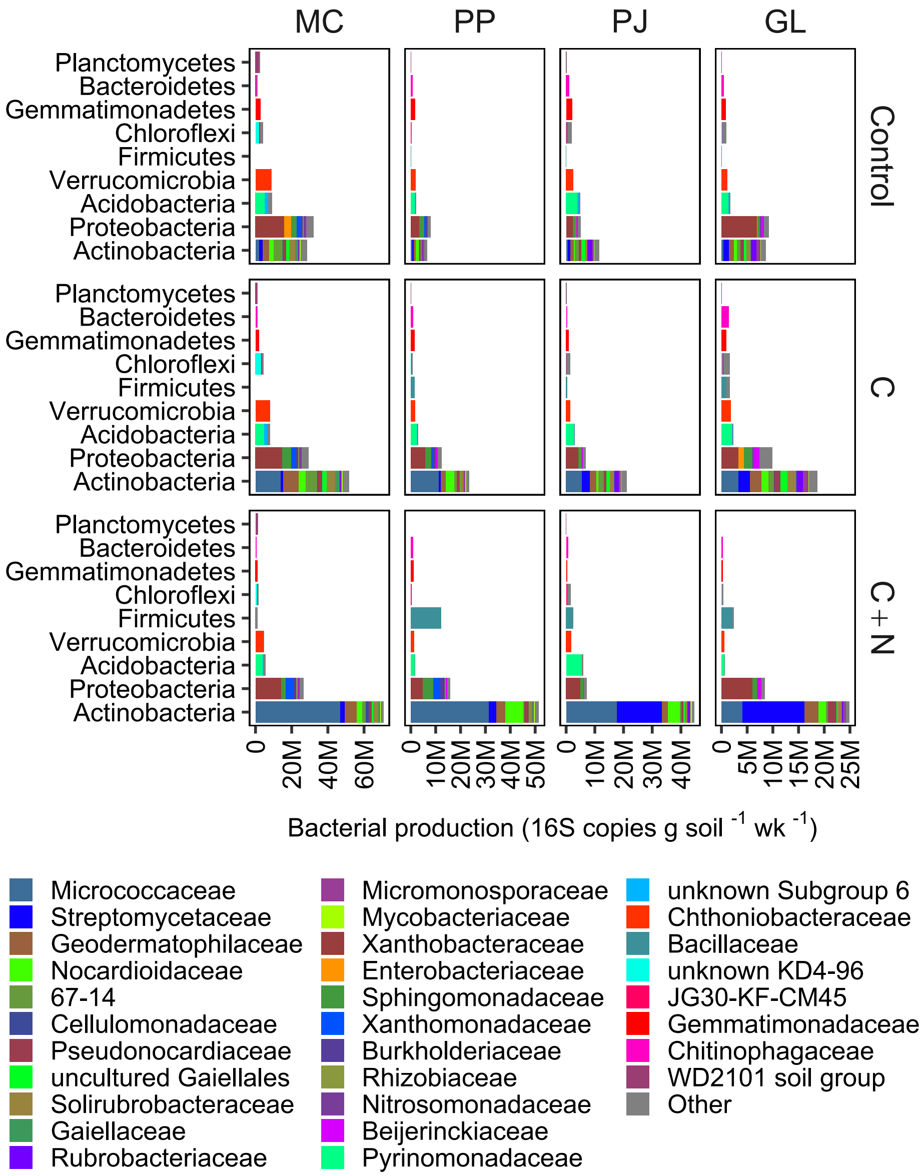

Supplementary Figure 3 Taxonomic profile of new 16S copies produced during a seven-day incubation. Values represent averages across replicates for each ecosystem (MC = mixed conifer forest, PP = ponderosa pine forest, PJ = piñon pine-juniper scrubland, GL = desert grassland) by treatment (Control = no amendment, C = glucose at 1000 μg C g^{-1} dry soil, C + N = glucose and nitrogen, $[NH_4]_2SO_4$, at 100 μg N g^{-1} dry soil) (n = 3). Stacked bars are colored by bacterial family (31 of 144 families shown, accounting for > 90% of new 16S production, remaining families colored gray and labeled as "Other"). Bars are grouped by the phyla that represent these 31 families.

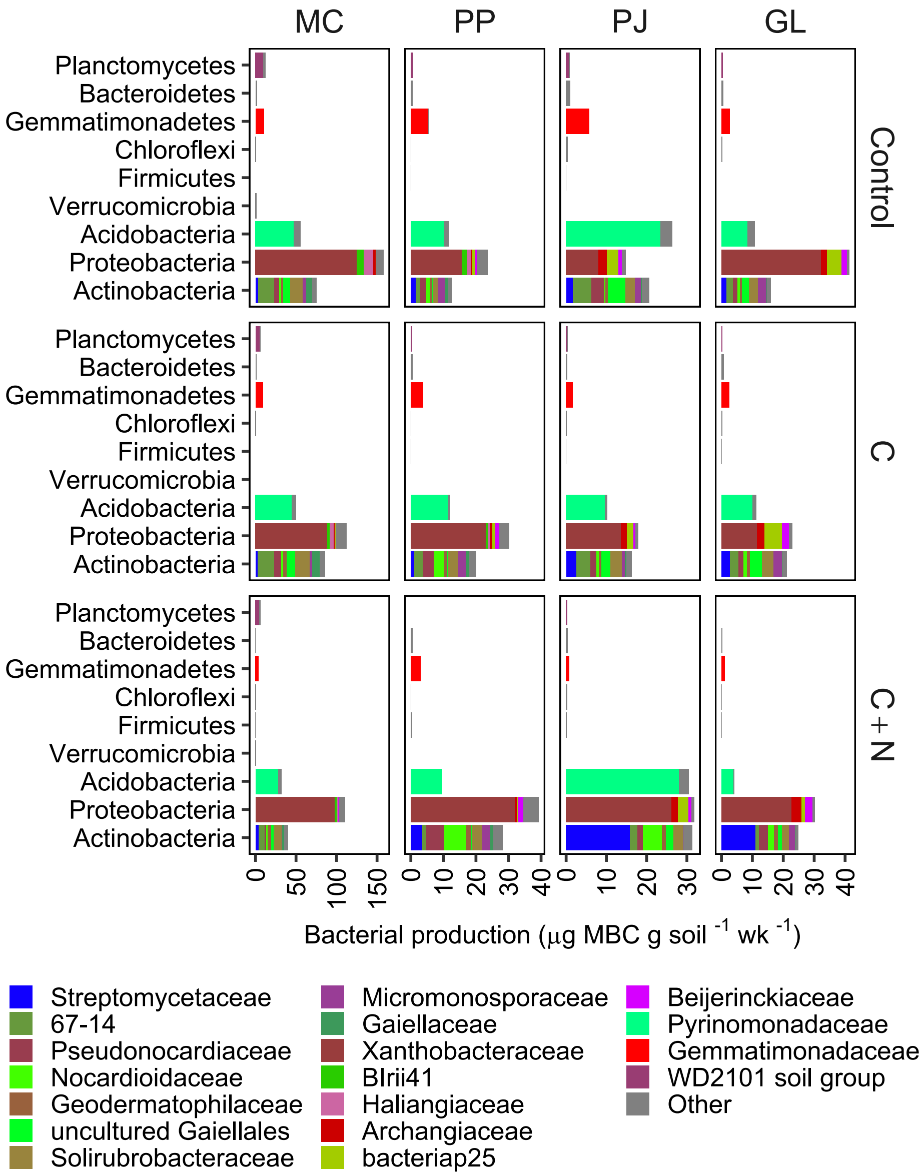

Supplementary Figure 4 Individual contributions to production Values represent averages of carbon produced as biomass (μg MBC g soil^{-1}) across replicates for each ecosystem (columns: MC = mixed conifer forest, PP = piñon pine-juniper scrubland, PJ = piñon pine-juniper forest, GL = desert grassland) by treatment (rows: Control = no amendment, C = carbon only, C + N = carbon and nitrogen amendment) combination (n = 3). Stacked bars and colored by bacterial family (19 of 144 families shown, accounting for > 90% of new biomass production, remaining families colored gray and labeled as "Other"). Bars are grouped by the phyla that represent these 19 families.

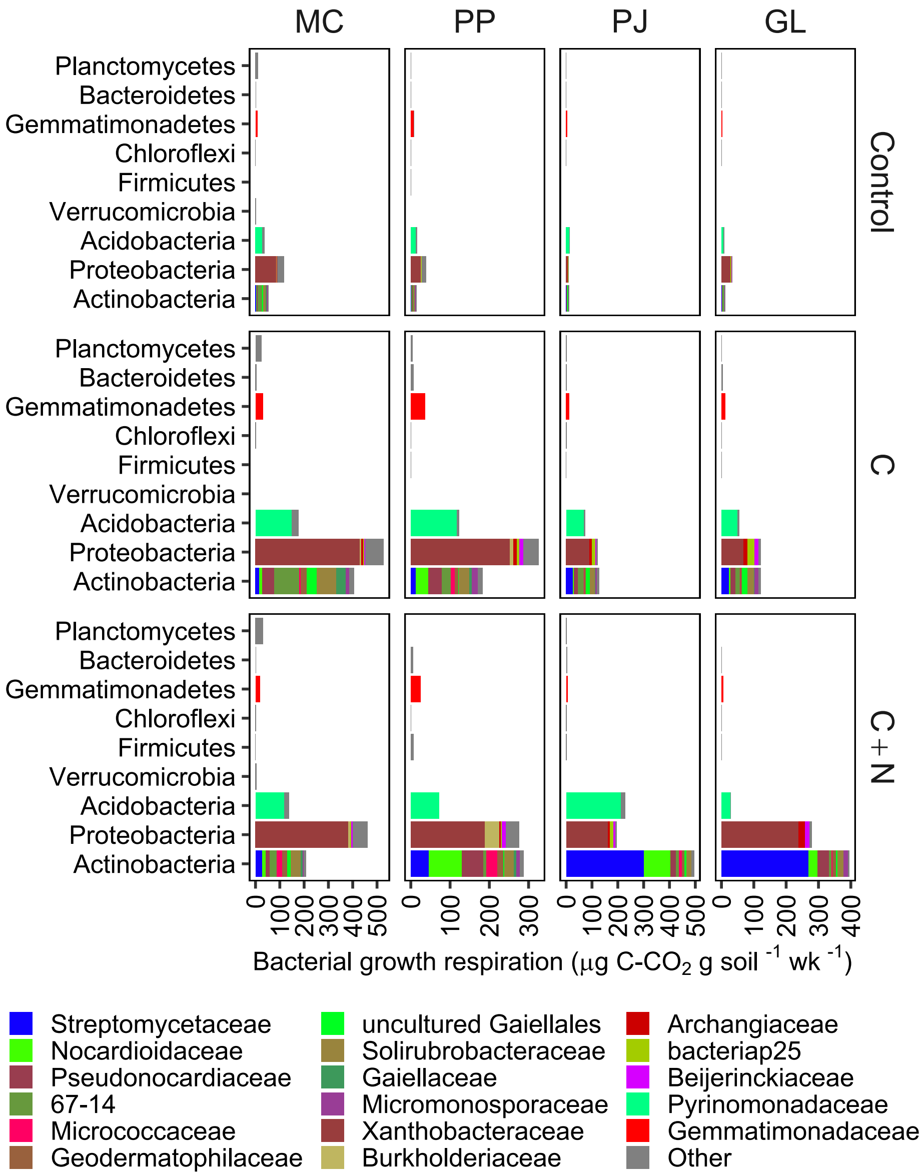

Supplementary Figure 5 Individual contributions to growth-related respiration. Values represent averages of carbon produced as CO_2 due to growth-related respiration (μg CO_2-C g soil^{-1}) across replicates for each ecosystem (columns: MC = mixed conifer forest, PP = ponderosa pine forest, PJ = piñon pine-juniper scrubland, GL = desert grassland) by treatment (rows: Control = no amendment, C = carbon only, C + N = carbon and nitrogen amendment) combination (n = 3). Stacked bars and colored by bacterial family (18 of 144 families shown, accounting for > 90% of new biomass production, remaining families colored gray and labeled as "Other"). Bars are grouped by the phyla that represent these 18 families.

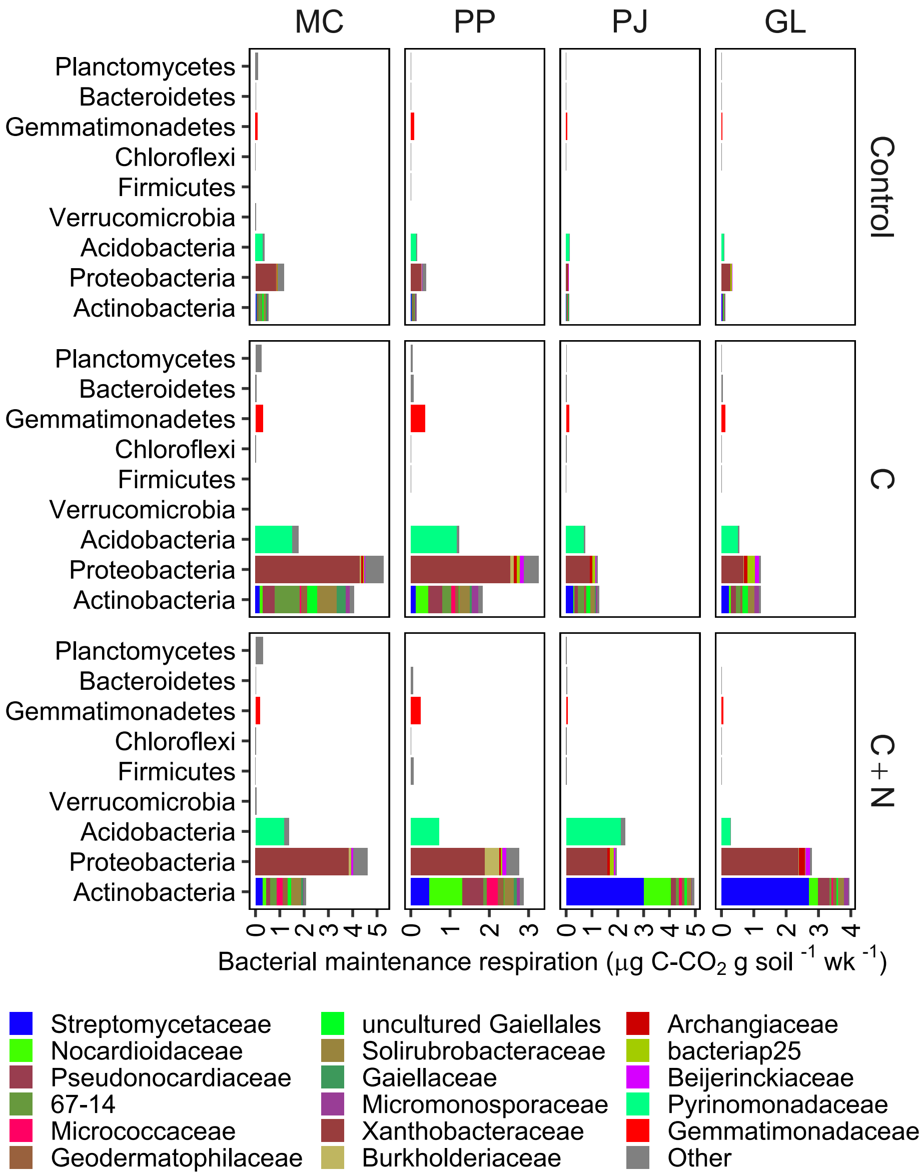

Supplementary Figure 6 Individual contributions to maintenance-related respiration. Values represent averages of carbon produced as CO_2 due to maintenance-related respiration (μg CO_2-C g soil^{-1}) across replicates for each ecosystem (columns: MC = mixed conifer forest, PP = ponderosa pine forest, PJ = piñon pine-juniper scrubland, GL = desert grassland) by treatment (rows: Control = no amendment, C = carbon only, C + N = carbon and nitrogen amendment) combination (n = 3). Stacked bars and colored by bacterial family (18 of 144 families shown, accounting for > 90% of new biomass production, remaining families colored gray and labeled as "Other"). Bars are grouped by the phyla that represent these 18 families.
