# Supplementary material for: Nutrients cause consolidation of soil carbon flux to small proportion of bacterial community
Source: Nat Commun. 2021 Jun 7;12:3381. doi: 10.1038/s41467-021-23676-x (PMC8184982; doi:10.1038/s41467-021-23676-x)
Supplement: Supplementary file 3 — Reporting Summary [file 41467_2021_23676_MOESM3_ESM.pdf]

nature research

Corresponding author(s): Bram Stone

Last updated by author(s): Apr 30, 2021

Reporting Summary

Nature Research wishes to improve the reproducibility of the work that we publish. This form provides structure for consistency and transparency in reporting. For further information on Nature Research policies, see our Editorial Policies and the Editorial Policy Checklist.

Statistics

For all statistical analyses, confirm that the following items are present in the figure legend, table legend, main text, or Methods section.

n/a	Confirmed	
☐	☒	The exact sample size (*n*) for each experimental group/condition, given as a discrete number and unit of measurement
☐	☒	A statement on whether measurements were taken from distinct samples or whether the same sample was measured repeatedly
☐	☒	The statistical test(s) used AND whether they are one- or two-sided<br>*Only common tests should be described solely by name; describe more complex techniques in the Methods section.*
☒	☐	A description of all covariates tested
☐	☒	A description of any assumptions or corrections, such as tests of normality and adjustment for multiple comparisons
☐	☒	A full description of the statistical parameters including central tendency (e.g. means) or other basic estimates (e.g. regression coefficient) AND variation (e.g. standard deviation) or associated estimates of uncertainty (e.g. confidence intervals)
☐	☒	For null hypothesis testing, the test statistic (e.g. F, t, r) with confidence intervals, effect sizes, degrees of freedom and P value noted<br>*Give P values as exact values whenever suitable.*
☒	☐	For Bayesian analysis, information on the choice of priors and Markov chain Monte Carlo settings
☐	☒	For hierarchical and complex designs, identification of the appropriate level for tests and full reporting of outcomes
☐	☒	Estimates of effect sizes (e.g. Cohen's d, Pearson's r), indicating how they were calculated

Our web collection on statistics for biologists contains articles on many of the points above.

Software and code

Policy information about availability of computer code

Data collection	BioTek Synergy HTX microplate reader software (version 2.04), Bio-Rad CFX Manager (version 1.0), Illumina MiSeq software (version 2.4)
Data analysis	Raw sequence data generated by Illumina MiSeq sequencing were processed using QIIME2 release 2018.6 which utilized DADA2 version 1.8 (https://www.github.com/benjjneb/dada2). Data and statistical analyses were conducted in R version 3.5.1. Digitization of figures utilized DataThief III release 1.5 (https://datathief.org/).<br><br>All data and code have been made publicly available on the author's Github page (https://github.com/bramstone/bacterial-carbon-flux-qSIP) with the doi 10.5281/zenodo.4592585

For manuscripts utilizing custom algorithms or software that are central to the research but not yet described in published literature, software must be made available to editors and reviewers. We strongly encourage code deposition in a community repository (e.g. GitHub). See the Nature Research guidelines for submitting code & software for further information.

Data

Policy information about availability of data

All manuscripts must include a data availability statement. This statement should provide the following information, where applicable:

- Accession codes, unique identifiers, or web links for publicly available datasets
- A list of figures that have associated raw data
- A description of any restrictions on data availability

All data and code have been made publicly available on the author's Github page (https://github.com/bramstone/bacterial-carbon-flux-qSIP) and accessible under doi 10.5281/zenodo.4592585

Field-specific reporting

Please select the one below that is the best fit for your research. If you are not sure, read the appropriate sections before making your selection.

☐ Life sciences ☐ Behavioural & social sciences ☒ Ecological, evolutionary & environmental sciences

For a reference copy of the document with all sections, see nature.com/documents/nr-reporting-summary-flat.pdf

Ecological, evolutionary & environmental sciences study design

All studies must disclose on these points even when the disclosure is negative.

Study description	Soil cores were collected from four sites along an elevation gradient in Northern Arizona and subject to parallel incubations of 13C or 18O and with the addition of either glucose or glucose plus ammonium sulfate to test microbial response to nutrient input. Following a 7 day incubation, a combination of buoyant density fractionation and 16S amplicon sequencing allowed for the estimation of microbial growth rates on a per-taxon basis. These per-taxon measures were subject to modeling in order to approximate individual contributions to microbial biomass productivity or respiration.
Research sample	The research sample was the soil bacterial community collected from 2 kg soil samples, as identified through 16S rRNA gene amplicon sequencing of the V4 hypervariable region of the gene. Samples were taken from the top 10 cm of soil and in plant-free locations. The samples were meant to represent the microbial population in soils at four distinct ecosystems. The rationale for this sampling scheme was that soil communities would be distinct based on the long-term differences in temperature and precipitation across different ecosystems. Samples taken closer to plant roots may have been affected by plant root exudates, potentially altering the community composition and function. Samples taken from deeper in the soil profile may less exposed to differences in precipitation and temperature, may be more influenced by the shared geological history and mineral composition across sites, and thus may not be distinct between ecosystems.
Sampling strategy	Sample size and incubation times in this study were based on previous qSIP experiments. Three replicates of 2 kg soil samples were collected from the top 10 cm in of plant-free patches in four ecosystems along the C. Hart Merriam elevation gradient in Northern Arizona beginning at high desert grassland (1760 m), and followed at higher elevations by piñon-pine juniper woodland (2020 m), ponderosa pine forest (2344 m), and mixed conifer forest (2620 m). Soils were air-dried for 24 h at room temperature, homogenized, and passed through a 2 mm sieve before being stored at 4 ºC for another 24 h. 1 g of each sample was given water either alone or in combination with 18O or with 13C in order to determine the isotope uptake and growth patterns of the microbial community in each situation. Sample sizes of three replicates per treatment and per ecosystem were chosen because this allowed for the calculation of measures of spread (variation). More replicates were not used due to the cost of parallel isotope incubations (including isotope-free incubations for control purposes, 18O, and 13C) and the fact that each single sample needed to be divided into 15-18 fractions, and amplified and sequenced separately, to quantify the assimilation of stable isotopes by individual bacterial taxa.
Data collection	Sampling and data collection (below) performed by authors RLM and MH.<br>Soil incubations were performed on soils with mass of 20 g of dry soil for measurements of CO2 and microbial biomass carbon (MBC), while 2 g of dry soil aliquots were incubated separately (but under equivalent conditions) for quantitative stable isotope probing (qSIP). We applied three treatments to these soils through the addition of water (up to 70% water-holding capacity): water alone (control), with glucose (C treatment; 1000 µg C g-1 dry soil), or with glucose and nitrogen (C + N treatment; [NH4]2SO4 at 100 µg N g-1 dry soil). All samples for qSIP were incubated with 18O enriched water (97 atom %) and matching controls necessary to calculate the change in 18O enrichment across the microbial community. We applied water at natural abundance (i.e., no 18O enriched water) to the larger soil samples prepared for measurement of carbon flux. All soils were incubated in the dark for one week. Following incubation, soils were frozen at -80 ºC for one week prior to DNA extraction. Soil, CO2, and microbial biomass measurements. We analyzed headspace gas of soils for CO2 concentration and δ13CO2 three times during the week-long incubation using a LI-Cor 6262 (LICor Biosciences Inc. Lincoln, NE, USA) and a Picarro G2201 (Picarro Inc., Sunnyvale, CA, USA), respectively. Prior to incubation we analyzed soil MBC using chloroform-fumigation extraction method on 10 g of soil. One sub-sample was immediately extracted with 25 ml of a 0.05M K2SO4 solution, while a second sub-sample was first fumigated with chloroform (for 5 days), after which it was similarly extracted. Following K2SO4 addition, we agitated soils for one hour, filtered the extract through a Whatman #3 filter paper, and dried the filtered solution (60ºC, four days). Salts with extracted C were ground and analyzed for total C on a Carlo Erba NC2100 elemental analyzer configured to a Delta V isotope ratio mass spectrometer (Thermo Fisher Scientific, West Palm Beach, Florida, USA). MBC was calculated as the difference between the fumigated and immediately extracted samples' soil C using an extraction efficiency of 0.45 (as per Liu et al.31).
Timing and spatial scale	Sample sites along the ecosystem gradient were separated by ~ 10 km, totalling ~30 km across the gradient. At each ecosystem, the triplicate samples were collected randomly within a 100 m-squared plot with at least 5 m between samples. Samples were taken October, 2014. The time scale of laboratory incubations was a 7 day incubation from July 18, 2014 to July 24, 2014 in the laboratory, samples were collected at time 0 and at the end of the incubation for destructive measurements. CO2 concentration and δ13CO2 was measured three times during the week-long incubation at 0, 48, and 72 hours.
Data exclusions	No data were excluded from the analyses.
Reproducibility	So far, there have been no attempts to reproduce the experiment. However, for future reproduction efforts, all code and data have been made available in publicly accessible repositories.
Randomization	Each replicate from each treatment was split and subject to each treatment level.

Blinding	Blinding was not done because aliquots of each field replicate (3 per ecosystem, across 4 ecosystems, 12 total) were subject to every isotope and nutrient treatment.

Did the study involve field work? ☒ Yes ☐ No

Field work, collection and transport

Field conditions	Ecosystems varied in their mean annual temperature (MAT) and precipitation (MAP). From high to low elevation, the sites included a mixed conifer forest (35.35° N, −111.73° W, MAT 4 °C, MAP 790 mm), a ponderosa pine forest (35.42° N, −111.67° W, MAT 5.5 °C, MAP 660 mm), a pinyon-juniper woodland (35.50° N, −111.62° W, MAT 7 °C, MAP 380 mm) and a high-desert grassland (35.58° N, −111.57° W, MAT 8.5 °C, MAP 230 mm).
Location	Mixed conifer forest (35.35° N, −111.73° W; elevation 2620 m), a ponderosa pine forest (35.42° N, −111.67° W; elevation 2344 m), a pinyon-juniper woodland (35.50° N, −111.62° W; elevation 2020 m) and a high-desert grassland (35.58° N, −111.57° W; elevation 1760 m).
Access & import/export	Samples were collected at a long-term elevation gradient field site with minimal disturbance to the surrounding area.
Disturbance	Disturbance was limited to the loss of ~6 kg of topsoil soil per site (minimal disturbance).

Reporting for specific materials, systems and methods

We require information from authors about some types of materials, experimental systems and methods used in many studies. Here, indicate whether each material, system or method listed is relevant to your study. If you are not sure if a list item applies to your research, read the appropriate section before selecting a response.

Materials & experimental systems

n/a	Involved in the study	
☒	☐	Antibodies
☒	☐	Eukaryotic cell lines
☒	☐	Palaeontology and archaeology
☒	☐	Animals and other organisms
☒	☐	Human research participants
☒	☐	Clinical data
☒	☐	Dual use research of concern

Methods

n/a	Involved in the study	
☒	☐	ChIP-seq
☒	☐	Flow cytometry
☒	☐	MRI-based neuroimaging

April 2020
